# Impacts of climate variability and future climate change on harmful algal blooms and human health

**DOI:** 10.1186/1476-069X-7-S2-S4

**Published:** 2008-11-07

**Authors:** Stephanie K Moore, Vera L Trainer, Nathan J Mantua, Micaela S Parker, Edward A Laws, Lorraine C Backer, Lora E Fleming

**Affiliations:** 1 School of Oceanography, University of Washington, Box 355351, Seattle, Washington 98195-5351, USA; 2 NOAA, Northwest Fisheries Science Center, West Coast Center for Oceans and Human Health, 2725 Montlake Blvd. E., Seattle, Washington 98112-2013, USA; 3 Climate Impacts Group and School of Aquatic and Fishery Sciences, University of Washington, Box 355020, Seattle, Washington 98195-5020, USA; 4 Pacific Northwest Center for Human Health and Ocean Studies, University of Washington, Box 357940, Seattle, Washington 98195-7940, USA; 5 School of the Coast and Environment, 1002 K Energy, Coast and Environment Building, Louisiana State University, Baton Rouge, Louisiana 70803-4110, USA; 6 National Center for Environmental Health, Centers for Disease Control and Prevention, 4770 Buford Highway NE MS F-46, Chamblee, Georgia 30341-3717, USA; 7 Department of Epidemiology and Public Health and Department of Marine Biology and Fisheries, University of Miami School of Medicine and Rosenstiel School of Marine and Atmospheric Sciences, 1120 NW 14th Street, Miami, Florida 33136-2107, USA

## Abstract

Anthropogenically-derived increases in atmospheric greenhouse gas concentrations have been implicated in recent climate change, and are projected to substantially impact the climate on a global scale in the future. For marine and freshwater systems, increasing concentrations of greenhouse gases are expected to increase surface temperatures, lower pH, and cause changes to vertical mixing, upwelling, precipitation, and evaporation patterns. The potential consequences of these changes for harmful algal blooms (HABs) have received relatively little attention and are not well understood. Given the apparent increase in HABs around the world and the potential for greater problems as a result of climate change and ocean acidification, substantial research is needed to evaluate the direct and indirect associations between HABs, climate change, ocean acidification, and human health. This research will require a multidisciplinary approach utilizing expertise in climatology, oceanography, biology, epidemiology, and other disciplines. We review the interactions between selected patterns of large-scale climate variability and climate change, oceanic conditions, and harmful algae.

## Introduction

Global atmospheric concentrations of carbon dioxide, methane, and nitrous oxides have increased markedly as a result of human activities since 1750, and now far exceed pre-industrial values determined from ice cores spanning many hundreds of thousands of years. Carbon dioxide (CO_2_) concentrations have increased from roughly 280 ppmv in pre-industrial times to present day levels of ~380 ppmv, mostly due to fossil fuel burning and deforestation [1]. The direct and indirect impacts of these increases in greenhouse gas concentrations on the oceans will include increasing temperatures, acidification, changes to the density structure of the upper ocean which will alter vertical mixing of waters, intensification/weakening of upwelling winds, and changes to the timing and volume of freshwater runoff into coastal marine waters, to name a few. Indeed, evidence is emerging to suggest that some of these changes are already underway [2].

The predicted changes in our oceans are likely to impact both directly and indirectly the interactions between humans and the oceans. Recent studies have reviewed general oceanic responses to future climate change, while acknowledging the impacts that these changes will have on human societies [3-5]. Likewise, over the past decade, several studies have suggested possible relationships between climate and the magnitude, frequency, and duration of harmful algal blooms (HABs) [6-11].

The term "harmful" has been used to describe blooms of algae that can cause a range of deleterious physiological and environmental effects [12]. Some harmful algae (HA) produce potent natural toxins that are bioconcentrated by some filter feeding shellfish and finfish, and passed along the food chain causing illness or death if consumed by humans or other organisms. Other HA are non-toxic, but attain high biomass resulting in substantial reductions in biodiversity of the phytoplankton community structure and the amount of light reaching the benthos [13]. The decomposition of senescent blooms can lead to serious reductions in dissolved oxygen concentrations. Because of the ecological and physiological diversities among HA species (discussed below), their responses to the same changes in climate will vary.

Few studies have used comprehensive datasets together with rigorous statistical analyses to confirm linkages between climate and HABs. Even fewer have attempted to include epidemiological information on HAB-related illnesses. The aim of this paper is to provide a synopsis of the current state of knowledge of climate impacts on HABs and their known and potential consequences for human health, particularly as it relates to the main research themes of the NSF/NIEHS and NOAA Centers for Oceans and Human Health.

We use the term "climate" here to refer to large-scale and low frequency patterns of climate variability, such as the El Niño/Southern Oscillation, as well as anthropogenic climate change. This is opposed to "weather" events that occur over much shorter timescales (i.e., days to weeks). It has been said of infectious disease that climate constrains the range and weather determines the timing and intensity of outbreaks [14]. This is also likely applicable to HABs. In this review, we summarize established climate impacts on the oceans and toxin-producing HA species, and discuss the implications for human health. Finally, directions for future research are provided.

This paper does not aim to prove that anthropogenic climate change is responsible for the global outbreak of HABs [15]. Few examples in the literature have quantitatively demonstrated this potential link; primarily because it is extremely difficult to separate the influence of climate change (natural and anthropogenic) from other anthropogenic impacts that are known to contribute to HABs using the limited datasets that are currently available. Nevertheless, evidence that climate change has influenced the frequency, duration, and geographical range of HABs is emerging as monitoring data temporally and spatially accumulates. Given the potential impact it is recommended that the influence of climate be considered and incorporated into future HAB research and monitoring efforts.

## Discussion

### Climate impacts on HABs

HA species comprise only a small component of the phytoplankton community, and their individual responses to climate variation and change can differ from that of the phytoplankton community as a whole. In the ocean, the most important HA and their poisoning syndromes (in parentheses) are diatoms from the genus *Pseudo-nitzschia *(amnesic shellfish poisoning), and species of dinoflagellates from the genera *Alexandrium, Pyrodinium*, and *Gymnodinium *(paralytic shellfish poisoning), *Karenia *(neurotoxic shellfish poisoning, and aerosolized Florida red tide respiratory syndrome), *Dinophysis *and *Prorocentrum *(diarrhetic shellfish poisoning), and *Gambierdiscus *(ciguatera fish poisoning). In freshwater, the most important HABs are caused by certain species of cyanobacteria (blue green algae) from the genera *Anabaena*, *Microcystis*, and *Aphanizomenon *(cyanobacterial poisoning) [16]. In both marine and freshwater systems, for humans and other animals, exposure to HA toxins results from eating contaminated fish or shellfish, drinking contaminated water, inhaling contaminated aerosol, or by contacting contaminated water.

It is generally accepted that HABs are increasing in frequency, intensity, and duration in all aquatic environments on a global scale [17,18], and some of this may be due to changes in climate. However, relatively little work has been done to characterize this link, and it is poorly understood [19-21]; primarily due to difficulty in separating the influence of climate change (natural and anthropogenic) from other anthropogenic impacts (such as eutrophication and ballast water introduction) that are known to contribute to the occurrence of some HABs. As time series of monitoring data lengthen, our ability to quantitatively determine climate change impacts on HABs will improve; but presently we are limited to relying on only a small number of studies.

The ocean is a core component of the climate system, such that perturbations to climate regimes can cause dramatic shifts in the structure and function of marine ecosystems [22-24]. This is demonstrated by examining the ecosystem responses to phase shifts of large-scale patterns of climate variability, such as the El Niño/Southern Oscillation and the Pacific Decadal Oscillation (ENSO and PDO, respectively). Both ENSO and PDO have warm and cool phases that typically last for 6 to 18 months and 20 to 30 years, respectively [25]. During warm phases, sea surface temperatures in the eastern and equatorial Pacific Ocean are anomalously warm, stratification is enhanced, and upwelling of nutrient-rich water along the eastern Pacific coast is reduced [26,27]. Given that phytoplankton growth is strongly determined by temperature, light, and the availability of nutrients, it is not surprising that these climate-ocean interactions result in changes to the phytoplankton community, and can influence HAB occurrence/development e.g. [28].

Ecosystem responses to these large-scale patterns of climate variability are not always consistent. For example, in the tropical Pacific Ocean the ecosystem response depends in part on whether the PDO and ENSO are in phase [29]. Nevertheless, reviewing the observed and modeled responses of ocean conditions and phytoplankton to the warm phases of large-scale climate variations is useful when trying to predict the likely impacts of future climate change. These studies suggest that the warmer upper ocean temperatures predicted under future climate scenarios will increase stratification of the water column and differentially influence phytoplankton growth in the global oceans [23,30-34]. In the thermally stratified oceans of the tropics and mid-latitudes, increased stratification and reduced vertical mixing decreases the nutrient supply to the surface, causing reductions in overall phytoplankton growth and biomass [23]. In these regions, organisms with lower nutrient requirements, or those having the ability to vertically migrate to nutrient-rich regions, will be favored [35]. At higher latitudes (in the polar and sub-polar salt-stratified oceans), phytoplankton growth and biomass are expected to increase. In these regions, nutrients are relatively abundant and phytoplankton growth is typically limited by light. Increased stratification due to the warming and freshening of the surface layer at high latitudes from melting ice will reduce vertical mixing and favor the retention of phytoplankton in the illuminated upper water column [31].

Phytoplankton need to remain close to the ocean's surface in order to capture sunlight for photosynthesis. If the surface becomes depleted of nutrients required for growth, certain types of phytoplankton will be favored. For example, most marine HABs are dinoflagellates, which are distinguished by the presence of two flagella used for swimming. Other phytoplankton groups, such as the diatoms, do not possess this swimming ability, and therefore do not have the potential to forage for nutrients deeper in the water column. Nutrients in the surface layer of the water column can become limiting by the combination of uptake by phytoplankton and the decrease in upward mixing of nutrients under stratified water column conditions. The swimming ability of dinoflagellates allows them to swim below the upper stratified layer of the water column to utilize nutrients in the deeper layer that other phytoplankton cannot access [36]. Dinoflagellates are therefore expected to be favored over other phytoplankton in marine environments under future climate scenarios. Assuming that dinoflagellate HA are favored by a more thermally-stratified ocean in the same way as other dinoflagellates, it is likely that the frequency of marine dinoflagellate HABs will increase as a result of climate change. However, more research is required to ascertain the response of dinoflagellate HA species to thermal stratification.

Warmer temperatures may result in expanded ranges of warm water HA species. For example, the tropical marine dinoflagellate, *Gambierdiscus toxicus*, is associated with ciguatera fish poisoning and primarily occurs as an epiphyte on some macroalgae. The abundance of *G. toxicus *correlates positively with elevated sea surface temperature during the warm (El Niño) phases of the ENSO cycle [37,38], and its range may extend to higher latitudes as temperatures rise due to climate change [39]. Indirect impacts of climate change may also cause incidents of ciguatera fish poisoning to become more frequent and more geographically widespread. For example, perturbations to coral reefs, such as hurricanes or bleaching events caused by increased water temperatures, free up space for macroalgae to colonize. Climate change impacts are predicted to increase the intensity of hurricanes and water temperatures [40], and may therefore increase habitat for *G. toxicus*.

**Figure 1:**
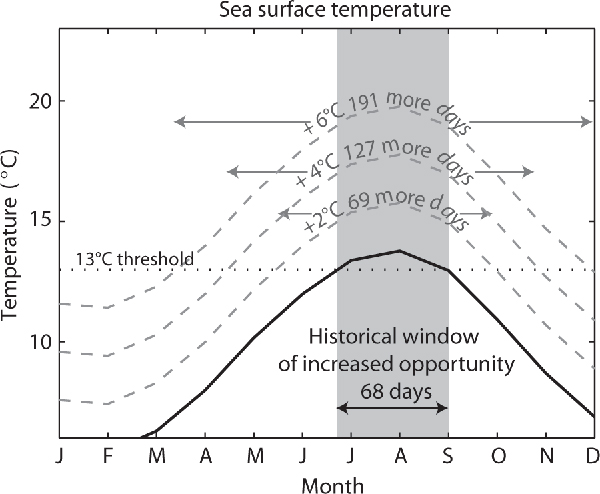
**Potential climate change impacts on Puget Sound shellfish toxicity**. Climatological monthly means of reconstructed sea surface temperature (SST) in Sequim Bay, Puget Sound, using detrended SST records at Race Rocks, British Columbia, from 1921 to 2007. The 13°C threshold for accelerated growth of *Alexandrium catenella *[42] is shown, and the mean annual window of favorable SST conditions is shaded for present day conditions. Scenarios for warmer SST conditions by 2, 4, and 6°C are shown in gray with the associated widening of the window of increased opportunity for *A. catenella *growth.

The period of time that HABs occur annually may also expand as a result of climate change. For example, the planktonic dinoflagellate *Alexandrium catenella *is associated with paralytic shellfish poisoning [41]. Water temperatures greater than 13°C have been found to promote *A. catenella *blooms [42], and in Puget Sound (Washington State), shellfish toxicity from this species occurs primarily in the late summer and early fall when the water temperatures reach their seasonal maxima [43]. By the year 2100, surface air temperatures in the Puget Sound region are predicted to increase by up to 6°C [44]. Given the close correspondence between Puget Sound air and water temperatures [45], the annual window of warm water temperatures exceeding 13°C will expand greatly (Figure 1). Optimal growth periods for freshwater HA may also expand as a result of warmer temperatures predicted under future climate scenarios, potentially favoring the growth of harmful cyanobacteria over other phytoplankton species [46]. Predicted rising water temperature may therefore promote earlier and longer lasting HABs [43]; however, it is important to acknowledge that interactions with other physical and biological aspects of the marine ecosystem will also influence the ultimate growth responses of HA species.

Changes in seawater CO_2 _concentrations and ocean acidity are also likely to influence phytoplankton species assemblages. The accumulation of CO_2 _in the atmosphere due to anthropogenic activities has increased concentrations of seawater CO_2 _and bicarbonate (HCO_3_^-^); both of which are inorganic carbon sources that can be utilized by phytoplankton for photosynthesis and growth. However, intrinsically linked with this change in seawater carbon chemistry is a decrease in ocean pH; a drop of roughly 0.1 units from pre-industrial levels has already been observed [2,47-49]. If anthropogenic CO_2 _emissions continue unabated, ocean pH could drop by an additional 0.6 units to a level lower than has occurred in the past 300 million years [40,49], with tropical regions and the Northern and Southern Oceans predicted to be impacted most severely [47,50].

Changes in phytoplankton species assemblages in response to increased dissolved CO_2 _and HCO_3_^- ^concentrations may result from enhanced growth of certain species [51], and/or from the inhibited growth of calcifying phytoplankton due to the dissolution of their biogenic calcium carbonate (CaCO_3_) shells [52], among other contributing factors [e.g., [48]]. How these changes will interact with other effects of climate change (such as warmer temperatures) to influence phytoplankton growth and species assemblages, including the growth and relative abundances of HA species, remains unknown [48,53]. Furthermore, experimental studies of HA species have largely focused on the effects of elevated pH levels. These studies, which include laboratory, field, and marine enclosure experiments, generally find positive relationships between pH and growth or toxin production of these species [54-56]. However, it is uncertain if these relationships will hold at the lower end of the pH scale in a more acidic ocean.

Looking ahead several hundreds of years is obviously speculative, but there is general agreement that a warmer Earth will be associated with climatic conditions similar to those characterized by the Mesozoic era [57], when the geological record indicates that dinoflagellates and coccolithophorids were favored among the eukaryotic phytoplankton [36]. Coccolithophorids are not a harmful algal species. However, the fact that they secrete calcium carbonate tests will make their survival problematic if the pH of aquatic systems drops by 0.7 units or more [3]. By making the environment more challenging for coccolithophorid survival, the non-calcareous phytoplankton will be given an advantage. A more acidic environment would favor, among others, the dinoflagellates – the group of phytoplankton to which most HA belong. Clearly, this is an aspect of climate change impacts research that deserves greater attention.

In freshwater systems, cyanobacterial HABs may also be impacted by pH changes resulting from climate change. Empirically, most freshwater cyanobacteria, and in particular species associated with HABs, are poor competitors with other phytoplankton at low pH [58,59]. Although the mechanisms responsible for the shift in competitive advantage is somewhat controversial [60], the empirical results are unequivocal [61]. Based on the chemical composition of the Great Lakes [62] and assuming a constant total alkalinity, the increases in atmospheric CO_2 _concentrations projected by Caldeira and Wickett [49] would drop the pH of the Great Lakes by 0.6 to 0.8 during summer months. In the case of Lake Erie, for example, the summer pH would decrease from ~9.5 to 8.7. Such a change would likely reduce the incidence of cyanobacterial surface scums [59], one of the common manifestations of freshwater cyanobacterial HABs. However, it is unknown if this will counter the predicted increase in growth rates of cyanobacterial HABs in response to warmer temperatures and nutrient overenrichment of waters [46].

Wind-driven upwelling at coastal margins plays an important role in the development of some HABs (see [63] for a review). The timing and strength of coastal upwelling winds are among the factors that will be affected by climate change, and may therefore have implications for HABs [64]. Upwelling along eastern ocean boundaries is predicted to intensify as a result of climate change [65]. Observations of increased upwelling intensity in the California Current system over the past 30 years support this theory [66,67]. While speculation exists over whether this can be attributed to anthropogenic climate change [68], results from one modeling study support the association [69]. On the other hand, a recent study of the Iberian Current system off northwest Spain has found that both the intensity and duration of upwelling has decreased since 1966 [70]. This significantly increased the water renewal time of the Rías Baixas, which increased the number of days annually that mussel rafts are closed to extraction due to HABs consisting primarily of the marine dinoflagellate *Dinophysis*, some species of which produce the toxin okadaic acid that causes the syndrome diarrhetic shellfish poisoning in humans [70].

Changes in the timing and volume of streamflow in snowmelt-dominated river basins are among the most established predictions of climate change impacts on natural resource systems [71]. Even with a fixed amount of precipitation, rising surface air temperatures cause a reduction in the fraction of annual precipitation stored as snow, and a shift in the timing of runoff to earlier dates in the spring. Shifts in the timing of runoff into coastal estuaries fed by snowmelt-derived rivers will lead to changes in the timing and magnitude of stratification, nutrient loading, and turbidity related to freshwater inputs. The consequences of these timing shifts for HABs are unknown.

Among the studies that assess the potential impacts of future climate change on HABs are a series of batch culture laboratory experiments simulating projected increases in temperature and salinity-stratification in the coastal zone of the Netherlands for the year 2100 [72,73]. The growth rates of potentially harmful *Dinophysis *and *Prorocentrum *species were found to double compared to growth rates under present conditions, while non-HA did not experience increased growth rates. This suggests that the risk of HABs of these species may increase in the future. However, it is important to acknowledge the limitations of these studies; the experiments were conducted as single species batch cultures, and interactions among HA and other biological components of the ecosystem (such as bacteria, other phytoplankton, and zooplankton) were not considered. Consequently, it is difficult to extrapolate the results of these experiments to the real world. This highlights the need to conduct ecologically relevant experiments and maintain long term monitoring programs over significant time periods and geographic diversity to specifically assess climate change impacts on HA.

### Implications for human health

Although the adverse health effects from exposure to HA toxins has been known for decades (for some of the cyanobacterial toxins) or more (e.g., brevetoxins associated with Florida red tides), very few epidemiologic studies designed to systematically assess these effects have been done. This has hindered the Intergovernmental Panel on Climate Change (IPCC) projections of climate change impacts on HAB-related illnesses [74], and will also make the detection and quantification of climate change impacts on HAB-related illnesses difficult.

Probably the most widely studied HA toxins are the brevetoxins associated with Florida red tides. Brevetoxins are a group of polyether toxins that affect sodium channels and can induce bronchoconstriction in the mammalian respiratory tract. The effects of human exposure to aerosolized brevetoxins have been examined in three of the populations most likely to be susceptible (i.e., beach visitors during Florida red tides, full-time lifeguards who must stay at their posts even during Florida red tides, and people with asthma who are likely to be more susceptible to environmental respiratory irritants). These studies have shown that healthy people acutely exposed to aerosolized brevetoxins during Florida red tide events experience respiratory irritation that is reportedly relieved when they leave the beach [75,76]. By contrast, people with asthma are more susceptible to brevetoxins, and may experience both acute and more long-term detriments in pulmonary function [77,78]. In addition, many asthmatics report that they experience respiratory symptoms for days after exposure. Additional work by Kirkpatrick et al. [79] has shown that there are increased admissions to emergency rooms for respiratory complaints (including pneumonia and bronchitis) during Florida red tides compared to times when there are no red tides. If *K. brevis *responds to increased stratification and reduced nutrients in ways similar to the responses of other dinoflagellate species, the occurrence of HABs of *K. brevis *may increase as a result of climate change; thus, the number of people exposed to brevetoxins will increase.

In addition to the work on marine HA toxins, more epidemiologic evidence is being reported on the effects of freshwater HA toxins. For example, recent work by Backer et al. [80] has begun to assess the health effects from recreational exposure to aerosolized microcystins during blooms of *Microcystis aeruginosa *in small lakes. The investigators found very low concentrations of microcystin in the air (0.02 to 0.46 ng m^-3^) and water (2 to 5 μg L^-1^) when people participated in recreational activities that generated aerosols, such as water skiing, or riding personal water craft. However, there were no reported increases in symptoms or respiratory illness after these exposures.

The incidence of human syndromes associated with exposure to HA toxins will increase as HABs occur more frequently and over greater geographic areas due to climate change. If we are going to be in a position to assess whether the human health effects from HA are increasing as more people come in contact with HABs, it is critical that data are collected describing baseline frequencies of the human illnesses associated with HABs, including the shellfish poisonings, ciguatera fish poisoning, cyanobacterial illness, and respiratory irritation from Florida red tide. One way to address this is to support HAB-related disease surveillance, such as the Harmful Algal Bloom-related Illness Surveillance System (HABISS) created by the Centers for Disease Control and Prevention (CDC). This system will collect data on human illnesses, animal illnesses, and the characteristics of the blooms themselves. For example, HABISS is part of a recently established linked network of public health information coupled with exposure and disease surveillance and environmental monitoring for Florida red tide, with weekly reports available by phone and web [81,82]. HABISS is also currently being integrated into other HAB monitoring programs, for example in the Great Lakes, Gulf of Maine, Hawaii, and Washington State. Over time, HABISS will be able to be used to assess whether increased contact between HABs and people and animals has a substantial impact on the frequency of HAB-related illnesses in a warmer climate.

### Climate change mitigation and HABs

In response to increased atmospheric CO_2 _concentrations and the development of the Kyoto Protocol, several strategies have emerged to reduce CO_2 _emissions and remove CO_2 _from the atmosphere. Carbon emission reduction strategies include the introduction of new technologies such as electric and hybrid electric vehicles and fuel cell technology. Several entrepreneurs have emerged in the global carbon credit market with CO_2 _removal strategies that capitalize on the vastness of the oceans to store carbon. Carbon capture and storage approaches to mitigate climate change often focus on capturing CO_2 _from large point sources such as fossil fuel energy facilities. The CO_2 _is then transported (for example, by pipeline) to a storage site such as deep geological formations or the deep ocean. This approach usually involves costly compression of CO_2_.

An alternative to point source carbon capture and storage is the proposed use of ocean phytoplankton communities as natural carbon capturing and removal machinery. In this model, oceanic carbon uptake is stimulated by enhancing phytoplankton productivity (usually through iron fertilization, see below), drawing CO_2 _into the oceans and ultimately sequestering it in the deep ocean. Nearly two decades ago, Martin and Fitzwater [83] demonstrated that phytoplankton productivity in high-nitrate low-chlorophyll (HNLC) regions is limited by the availability of iron. Iron is an essential element for photosynthesis and many other biological processes, yet in many open ocean environments, surface iron concentrations are extremely low. Since this discovery, several large-scale iron fertilization experiments have confirmed the original hypothesis. In nearly every experiment, diatoms were among the dominant phytoplankton species to respond to iron enrichment (Table 1). Recent assessments suggest diatom-mediated export production can influence climate change through uptake and sequestration of atmospheric CO_2 _[84]. Thus, iron fertilization of the oceans has the potential to draw-down atmospheric CO_2 _levels by stimulating large blooms of diatoms.

**Table 1:** Dominant diatom genera in large-scale iron fertilization experiments

**Year**	**Experiment**	Dominant Phytoplankton*	**Diatom genera responding to Iron Enrichment**^§^
1993	IronEx	Mixed	*no data*
1995	IronExII	Diatoms	***Pseudo-nitzschia***^†^
1999	SOIREE	Diatoms	*Fragilariopsis, Thalassiosira, Rhizosolenia*, ***Pseudo-nitzschia***, *Nitzschia, Thalassiothrix*
2000	EisenEx	Diatoms	***Pseudo-nitzschia***, *Fragilariopsis*, *Thalassionema*, *Chaetoceros*, *Corethron*
2001	SEEDS	Diatoms	*Chaetoceros*, ***Pseudo-nitzschia***, *Rhizosolenia*, *Leptocylindrus*, *Eucampia*, *Neodenticula*
2002	SERIES	Diatoms	***Pseudo-nitzschia***, *Neodenticula*, *Thalassiothrix*, *Chaetoceros*, *Rhizosolenia*, *Thalassiosira*, *Proboscia*
2002	SOFeX (north)	Mixed	***Pseudo-nitzschia***
2002	SOFeX (south)	Diatoms	*Fragilariopsis, Corethron, Chaetoceros, Rhizosolenia*
2004	EIFEX	Diatoms	*Thalassiothrix, Corethron, Rhizosolenoids*, ***Pseudo-nitzschia***, *Fragilariopsis, Dactyliosolen*
2004	SEEDS II	Mixed	***Pseudo-nitzschia***, *Neodenticula*

Numerically dominant diatom genera in blooms induced by large-scale iron fertilization experiments. Experiments summarized here are the Iron Enrichment Experiments I and II (IronExI and IronExII), Southern Ocean Iron Release Experiment (SOIREE), Iron Experiment (EisenEx), Subarctic Pacific Iron Experiment for Ecosystem Dynamics Study (SEEDS), Subarctic Ecosystem Response to Iron Enrichment Study (SERIES), Southern Ocean Iron Experiments – North and South (SOFeX north and south), European Iron Fertilization Experiment (EIFEX), and Subarctic Pacific Iron Experiment for Ecosystem Dynamics Study II (SEEDS II). The genus *Pseudo-nitzschia *is in bold to highlight the ubiquity of this genus in large-scale iron enrichment experiments. *Data from Boyd *et al. *[100]. ^§^Data compiled from Marchetti *et al. *[101], Hoffmann *et al. *[102], Tsuda *et al. *[103] and [104], and Martin *et al. *[105], Landry *et al. *[106]. ^†^During IronExII, *Pseudo-nitzschia *was originally misidentified as *Nitzschia *(K. Coale, pers. comm.).

Subsequent studies, however, have begun to show that this approach is not as straightforward as it may seem [48]. For instance, the amount of atmospheric carbon that is exported to the deep ocean during iron fertilization experiments is much less than that predicted by early studies [85,86]. The conclusions have raised concerns about an attempt to use iron fertilization as a solution to the rise of atmospheric greenhouse gases [e.g. [87,88]]. Furthermore, the algae that respond to iron fertilizations in HNLC regions are often pennate diatoms that include *Pseudo-nitzschia *(Table 1), hypothesized to regulate the production of its potent neurotoxin, domoic acid, in response to levels of available iron and copper in seawater [89].

Recent research has uncovered a gene encoding an iron storage protein (ferritin) in several species of *Pseudo-nitzschia *[90]. Together with the finding that domoic acid (DA) can complex trace metals such as iron and copper [89,91], this may help to explain their success in HNLC regions. Ferritin is a common molecule in many organisms, but its presence in diatoms had not previously been detected. Preliminary results suggest that the gene for ferritin may be confined to only a small subset of diatoms that includes the HA genus *Pseudo-nitzschia *[90]. *Pseudo-nitzschia *commonly occur along coastlines and are responsible for closures of shellfish and finfish harvests due to accumulation of DA in the tissues of commercially and recreationally important marine species. Very few open ocean *Pseudo-nitzschia *species have been isolated into culture. Even fewer isolates have been tested for DA production, thus far with negative results, indicating that either *Pseudo-nitzschia *species from HNLC ocean environments produce little or no DA or that the unique culture conditions required to elicit toxin production were not met [92]. Given the success of *Pseudo-nitzschia *species in iron enrichment experiments, it is important to investigate further their potential to produce toxin to better evaluate the consequences of proposed large-scale ocean fertilization to mitigate increasing atmospheric carbon [92].

### Successes, challenges, and the way forward

**Figure 2:**
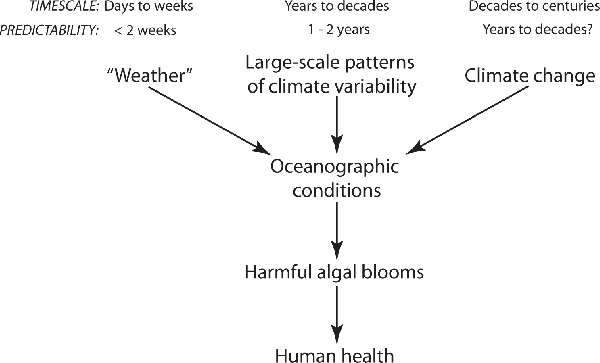
**Prediction timescales for climate impacts on HABs and human health**. Timescales and limits of prediction for atmospheric forcing of oceanographic conditions and harmful algal blooms and their impacts on human health. Aspects of the atmospheric climate are grouped into three categories; "weather", large-scale patterns of climate variability and climate change.

Interactions among the atmospheric climate, oceanic conditions, and HABs occur on a range of timescales, with implications for the accuracy of predictions of HAB risks and subsequent HAB-related illnesses in humans and other animals (Figure 2). Note that predicting HAB risks is fundamentally different from predicting HAB occurrence, with the former being an assessment of the likelihood or probability of the latter. Quantitative prediction of HAB initiation and distribution in space and time continues to elude scientists and resource managers. Accurate prediction will require a deeper understanding of HAB physiology and ecology than is presently known in order to adequately constrain parameters in coupled physical-biological models. However, if currently available information can be used to generate qualitative predictions of HAB risks, managers can be forewarned and operate at a heightened level of caution and can be prepared to respond rapidly if HAB risks are "high."

If local environmental factors are creating conditions favorable for HAB development, advanced warning of HAB risks will be limited by the fundamental predictability of the weather (i.e., up to 2 weeks) [93]. "Weather" events occur on a timescale of days to weeks and include local wind, precipitation and air temperature. Alternatively, large-scale patterns of climate variability (such as ENSO and PDO) can be predicted up to 1 year in advance [94,95]. Therefore, if oceanic conditions favorable for HAB development are well-correlated with variations in large-scale climate factors, as is the case for ENSO and *G. toxicus *which is associated with ciguatera fish poisoning [37,38], then advanced warning of HAB risks may be considerably extended in time.

One of the biggest challenges to research on climate change impacts on HAB variability and HAB-related illnesses is the paucity of long-term datasets with information in the necessary time and space scales to resolve key biophysical processes. The patchy and episodic nature of HABs begs for monitoring systems that provide continuous and co-located time series of physical, chemical, and biotic properties with at least daily observations. Specifically, this would include observations of phytoplankton species and concentration, nutrient and water chemistry profiles (CO_2 _and O_2_), temperature and salinity profiles, toxins, surface winds, and solar radiation [96]. Additional data from surveillance of HAB-related illnesses in humans and other animals would also be useful and would improve prediction capabilities [14]. Such historical databases and current operating observing systems are scarce [13,97].

Long time-series of observations are required to separate the influence of natural patterns of large-scale climate variability (such as ENSO and PDO) from anthropogenic climate change signals. Few observational and monitoring programs that track the chemical and/or biotic properties of marine (plankton) ecosystems have continuity over several decades. Those that have been sustained into the present from the early to mid 1900s have proved invaluable to climate research.

An example of a dataset which could be used to study global change and HABs in the future is the Continuous Plankton Recorder (CPR) Survey which provides near unbroken monthly coverage of transects across the northeast Atlantic and North Sea from 1946. Most of the region sampled by the CPR is further than 1 km offshore in open ocean waters, and is therefore relatively unaffected by anthropogenic eutrophication that is generally concentrated along the coast [11]. Even though most HA species are too small to be captured by the CPR, changes in the relative abundances of functional groups of phytoplankton (i.e., diatoms and dinoflagellates), and of some larger HA species, such as *Prorocentrum *and *Dinophysis *spp., can be assessed. Using this long-term dataset, the influence of the North Atlantic Oscillation (NAO) on the spatial distribution of these larger HA species was determined independently from the effects of anthropogenic eutrophication [11]. Distinguishing these effects lends credence to predictions of climate change impacts on HABs based on their response to warm phases of large-scale patterns of climate variability. Monitoring programs with comparable spatial and temporal resolution to the CPR, but specifically targeting smaller size fractions of plankton containing HA species, will be required to elucidate climate change impacts in the future.

In a 1994 review on the potential consequences of climate change for toxic HABs, Tester commented that "our research, monitoring, and regulatory infrastructure is not adequately prepared to meet an expanding global threat" [39]. More than a decade later, limited progress has been made towards minimizing this future threat to human health. Future research should focus on further elucidating relationships between HABs and aspects of the local and large-scale climate. Foremost, this will require the development and deployment of observing systems to provide the consistent, long-term data necessary to identify links between HABs and the suite of ecosystem variables that are important for HA variability. There is also a pressing need to engage the human health community to ensure that environmental and human data not only characterize HAB events, but can also be used to predict human health risks. Observational data must then be integrated into empirical, theoretical, and numerical simulation models to test and validate hypotheses.

The IPCC is now considering making HAB risk forecasts, and assessing the potential impacts of climate change on HABs under the suite of 21^st^-century climate change scenarios [1,13]. A crucial step in assessing questions of climate change impacts on HABs will be matching the appropriate scales of information. Presently, the IPCC has organized efforts to explore the impacts of increasing greenhouse gases and changing aerosol concentrations on large-to-global scale climate changes. Efforts to match the outputs from the global scale climate studies to much more localized climate change impacts studies must first elucidate the local processes that give rise to HAB variations, and then to develop new methods for downscaling the IPCC climate change scenarios that can be used to build the bridge to HAB impacts studies. Currently, typical IPCC global climate models provide information on a grid with ~100 to 300 km resolution, which may not prove to be fine enough to be useful for local scale (1 to 10 km) HAB forecasting and risk assessments.

The approach to downscaling future climate change projections should be guided by the mechanisms that are both (i) demonstrated to be important environmental links to HAB risks, and (ii) demonstrated to be well-informed by climate model outputs. For example, sea surface temperature in many estuaries is well correlated with regional surface air temperature, so statistical downscaling of temperature may prove useful in situations where HAB risks are clearly sensitive to surface water temperatures. In contrast, where local wind-forcing, stratification, and estuarine circulation are critical factors for HAB risks, it may be necessary to run much higher resolution (1 km) nested atmosphere and estuarine circulation models to assess the potential impacts of climate change on HAB risks. Whatever the case, efforts to match the outputs from global scale climate studies to more localized climate change impacts studies must first elucidate the local processes that give rise to HAB variations.

Finally, future research should focus on better elucidating relationships between HA and other biological components of the ecosystem. For example, interactions between the toxin-producing species of *Pseudo-nitzschia *and bacteria have been shown to influence production of domoic acid (see [13] and references therein). However, it is uncertain if and how anthropogenic climate change will influence this interaction. Similarly, physiological rates of uptake of HA-derived toxins by finfish and shellfish may change. Given the public uncertainty that already surrounds seafood consumption, recently termed the "seafood dilemma" [98], it will be important to monitor any increases in the susceptibility of seafood species to contamination by HABs, as well as the subsequent risks to consumers.

## Conclusion

Substantial research is needed to evaluate the direct and indirect associations between climate change, HABs, and human health. Specifically, future research efforts should focus on developing the empirical, theoretical, and numerical simulation models to integrate observations, test and validate hypotheses, and make risk forecasts of HAB occurrences and their impacts on human health under the suite of 21^st^-century future climate change scenarios. Such models will require simultaneous measurements of physical and biological environmental parameters made available through routine and consistent monitoring, and the identification of exposure health endpoints. To investigate this potential consequence of climate change, a new type of combined oceanographic-epidemiologic study that addresses very long time spans and extensive geographic scales will be needed [99].

## Competing interests

The authors declare that they have no competing interests.

## Authors' contributions

SKM took lead authorship of this manuscript, all other authors contributed equally. All authors read and approved the final manuscript.
